# Remission as an Emerging Therapeutic Target in Type 2 Diabetes in the Era of New Glucose-Lowering Agents: Benefits, Challenges, and Treatment Approaches

**DOI:** 10.3390/nu14224801

**Published:** 2022-11-13

**Authors:** Dimitra Vasdeki, Theocharis Koufakis, Georgios Tsamos, Luca Busetto, Pantelis Zebekakis, Kalliopi Kotsa

**Affiliations:** 1 Division of Endocrinology and Metabolism and Diabetes Center, First Department of Internal Medicine, Medical School, Aristotle University of Thessaloniki, AHEPA University Hospital, 54636 Thessaloniki, Greece; 2 Department of Medicine, University of Padova, 35121 Padova, Italy

**Keywords:** diabetes remission, diet, weight loss, bariatric surgery, diabetes pharmacotherapy

## Abstract

Type 2 diabetes mellitus (T2DM) is a progressive disease with a growing prevalence, associated with an increased risk of complications. The introduction of new classes of antidiabetic drugs into clinical practice has dramatically changed the landscape of diabetes therapy. However, despite the progress made in the pharmacotherapy of T2DM, mitigating the burden of the disease on individuals, societies and health care systems remains a challenge. Remission has recently emerged as a therapeutic target in T2DM, achievable through a wide range of interventions. Recent studies have shown that extensive lifestyle changes, such as weight reduction, bariatric surgery, and intensive glucose lowering therapy, can prompt the remission of diabetes, but some unanswered questions remain regarding its long-term effects on diabetic complications. Metabolic surgery and novel classes of glucose-lowering medications are currently the most effective interventions to induce weight loss and by extension remission in patients with diabetes; however, the ideal strategy to achieve the long-term maintenance of remission remains doubtful. In this narrative review, we discuss the available therapeutic approaches to target the remission of diabetes through personalized multimodal care, based on the latest evidence.

## 1. Introduction

### 1.1. Complex Problems Can Only Have Complex Solutions

Diabetes mellitus is a chronic disease with a complex pathophysiological background, associated with genetic and environmental factors. Key pathogenetic mechanisms include deficient insulin secretion, resistance to peripheral insulin actions, increased glucose reabsorption by the kidneys, increased liver glucose output, impaired incretin secretion, and neurotransmitter defects [1,2]. Chronic hyperglycemia is one of the main causes of diabetes complications, including cardiovascular events, kidney failure, and visual loss due to well-established microvascular and macrovascular impairment associated with diabetes [1]. Since 1980, there has been a dramatic increase in the prevalence of type 2 diabetes mellitus (T2DM), with recent data showing that 700 million people between 20 and 79 years of age will be affected by 2045 worldwide [3,4,5].

Well-defined risk factors for the development of T2DM include an increase in body mass index (BMI) and waist circumference, reflected in the abnormal accumulation of adipose tissue in central depots. Recent studies reported that in patients with T2DM, fluctuations in BMI are associated with a higher risk of mortality and cardiovascular events [6]. However, not all people affected by obesity will develop metabolic complications such as T2DM. The interaction between insulin resistance, adiposity, and β-cell function is variable, reflecting the heterogeneous clinical presentation and course of T2DM [7]. According to the most recent estimates, obesity rates increase by 3.09% per year in men and 1.92% per year in women, while in 2014, 66% of men and 55% of women in Greece were overweight (BMI ≥ 25 kg/m^2^) [8].

Diabetes is known to have an increased prevalence in ethnic minorities due to several factors, including low socioeconomic status, educational level and household income, emotional stress, and unhealthy eating patterns [9,10]. According to many studies, the prevalence of T2DM is lower in the highest income category compared to the lowest income category [11]. Especially in women, socioeconomic factors such as income, education level, and food insecurity appear to be independent risk factors for incident diabetes [12]. Lifestyle factors such as unhealthy diets, tobacco use, physical inactivity, and heavy alcohol consumption are correlated with elevated blood pressure, elevated serum cholesterol, and overweight [13]. These factors contribute to the early presentation of T2DM, increased cardiovascular risk, and therefore mortality [14]. Furthermore, occupational and emotional stress, depression, and sleep disorders related to circadian disturbances induce insulin resistance and inflammation, and eventually lead to an increased risk of diabetes [15]. Based on the above, it becomes evident that the complexity of factors that contribute to the development of T2DM requires interventions at the physiological, behavioral and social level to be effective.

### 1.2. Start Is Half of Everything: Defining Diabetes Remission

Until now, there has been no universal agreement on how remission should be defined. A very recent consensus report defined remission as glycated hemoglobin (HbA1c) <48 mmol/mol (6.5%) or fasting blood glucose <7 mmol/L (126 mg/dL), or estimated HbA1c less than 48 mmol/mol (6.5%) calculated from continuous glucose monitoring values, maintained for at least 3 months without any glucose-lowering pharmacotherapy [16]. Previous attempts to define remission adopted different approaches: in 2009, diabetes remission was classified into three distinct types—partial, complete, and prolonged remission. Specifically, partial remission was characterized as “sub-diabetic hyperglycemia” with a duration of at least one year in the absence of active pharmacologic therapy or ongoing procedures, complete was characterized as “a return to normal” measures of glucose metabolism with a duration of at least one year in the absence of active pharmacologic therapy or ongoing procedures, and prolonged was characterized as “complete remission” lasting more than 5 years [17]. A considerable number of studies claimed that remission of diabetes is possible through pharmacologic or surgical therapy combined with lifestyle approaches such as weight loss and intensive dietary changes [18,19,20]. However, the greatest challenge to achieve remission is maintaining long-term weight loss and lifestyle change. Therefore, while there are data supporting the suggestion that the remission of diabetes is now feasible, there are several barriers at the health system, physician, and patient level that make it difficult to achieve. This narrative review will analyze each of the available therapeutic approaches to induce remission, identifying the advantages, drawbacks, and challenges in pursuing this emerging target of diabetes management.

## 2. Methods of Literature Search

For this review, a comprehensive literature search was performed in the PubMed, Cochrane Library, and Google Scholar databases to identify relevant studies written in English. A combination of the following search terms was used: “remission”, “type 2 diabetes”,” reversal”, “remission induction”, “weight loss strategies”, “lifestyle”, “diet”, “anti-obesity agents”, “bariatric or metabolic surgery”, “physical activity” AND “limits specified” (human patients, English language). Additionally, references from the retrieved articles were searched to identify relevant works. Evidence from systematic reviews, meta-analyses, and published guidelines was also included. Case reports, protocols, and studies focusing on prediabetes, impaired glucose tolerance, impaired fasting glucose, gestational diabetes, maturity onset diabetes of the young, steroid-induced diabetes, or type 1 diabetes were excluded as beyond the scope of this narrative review. The final search was conducted in July 2022.

## 3. Therapeutic Approaches for Remission: Different Ways, Same Destination

### 3.1. Bariatric Surgery

More than 75% of patients with T2DM are estimated to be overweight, obese or have increased waist circumference, which is considered a reliable marker of metabolic and cardiovascular risk [21,22]. Bariatric surgery aims to modify the upper gastrointestinal tract (GIT) to treat obesity and its comorbidities and promote improvement in glycemic control. The role of GIT in the management of T2DM is gaining increasing attention [23,24]. However, the exact mechanisms by which bariatric surgery leads to remission of diabetes have not been fully understood. Several studies showed that in addition to weight loss, an improvement in glucose tolerance is observed, mediated by a dramatic improvement in insulin resistance of approximately 50% within one week after surgery. These benefits are only partially explained by a decrease in calorie intake, which in turn leads to a reduction in fat deposition in the liver [25,26,27]. Metabolic surgery results in changes in the hormonal environment, such as increased levels of glucagon-likepeptide-1 (GLP-1) and the YY peptide, both of which have been involved in weight loss. An increase in circulating bile acids has also been observed, translating into an alteration of the intestinal microbiome [28]. Several studies also claimed that higher levels of serum bile acids are associated with the stimulation of GLP-1 secretion. These combined effects can improve pancreatic β-cell function and lead to increased insulin release and sensitivity [29,30]. Bariatric surgery has also been shown to promote an alteration in the equilibrium of other GIT hormones, including ghrelin, oxyntomodulin, cholecystokinin, and obestatin, which are associated with the preservation of glucose homeostasis [31].

There are different types of bariatric surgery procedures, such as adjustable gastric band (AGB), biliopancreatic diversion (BPD), vertical sleeve gastrectomy (SG), and Roux-en-Y gastric bypass (RYGB), which generate different physiological results. Most studies showed that patients subjected to surgery were two-fold more likely to achieve remission of diabetes with RYGB than with AGB [32]. The most common metabolic surgery procedures are SG and RYGB. In the former, about 80% of the stomach portion is removed along the greater gastric curvature, leading to reduced stomach volume, which retains less food, and through hormonal changes, hunger is reduced and emptiness is delayed. In the latter procedure, the stomach is separated into a smaller pouch in the smaller curvature (through stapling) and anastomosed with the jejunum [33]. In this way, the gastric pouch remnant cannot hold a large amount of food and the procedure leads to alterations in the gut–brain axis that increase satiety and promote weight loss. However, not all patients with T2DM and obesity are suitable for a surgical ‘curative’ option. It would be of great importance to establish specific characteristics of the patient before surgery that could predict the probability of postoperative diabetes remission, such as age, BMI, duration of diabetes, etc.

Table 1 summarizes studies investigating the effectiveness of bariatric surgery in inducing diabetes remission. As shown, variable remission rates have been reported. In the study by Mingrone et al., the primary endpoint was diabetes remission in patients with T2DM following metabolic surgery versus pharmacotherapy, in which notable remission rates were reported with the former (75% in the gastric bypass group and 95% in the biliopancreatic diversion group) [34]. More recently, the same group published another study with a 10-year follow-up period, which showed that both surgical groups achieved high remission rates (58.8% relapsed during the follow-up period; however, they maintained their euglycemic status) [35]. In 2017, Schauer et al. provided long-term evidence (5 years of follow-up) on metabolic benefits in individuals who underwent surgical procedures and patients who received medical therapy, showing that the surgery group had a significantly higher mean percentage of reduction in HbA1c levels [36]. Young et al. reported that bariatric surgery had a significant long-term positive impact (mean follow-up 61 months) on body weight and albuminuria in patients with T2DM, in addition to improving glycemic control and inducing remission [37].

### 3.2. Dietary Interventions

Nutritional interventions play a key role in achieving glycemic control in people with T2DM by reducing the total calorie intake, energy absorption, or appetite [46]. Diets with different macronutrient composition, such as low-energy diets (LED) and low-carbohydrate diets (LCD), regulate glucose homeostasis through different mechanisms. LEDs typically provide 800–1200 kcal/d as a total or partial replacement for the typical diet [47,48]. In 2018, a trial showed that 46% of participants with T2DM achieved remission using total LEDs [48]. LCDs are defined as those providing <26% of total energy from carbohydrates (or 130 g/d), and ketogenic diets are defined as those providing <10% of the total energy from carbohydrates (or 50 g/d) [49]. Significant reductions in body weight, especially if they are greater than 15 kg compared to baseline weight, are highly predictive of remission in people with diabetes [48]. In 2011, Lim et al. showed that people on a very low-calorie diet (VLCD) displayed a reduction in fasting plasma glucose to non-diabetic levels, as well as serum triglycerides [47]. However, the meta-analysis of Korsmo-Haugen et al. demonstrated that VLCD did not lead to greater weight loss compared to carb-rich diets over a period of 3 to 36 months [50]. In general, trials investigating the effectiveness of VLCD in promoting remission present differences in their study design, including the duration of the intervention (8–20 weeks) and the calorie intake (510–853 kcal/day) [51]. In some studies, a significant proportion of patients who achieved remission (approximately 25%) regained their baseline weight, leading to relapse of diabetes by 24 months [18]. The available evidence indicates that the magnitude of weight loss (typically more than 15% of baseline weight), rather than the composition of the diet, is the strongest predictor of remission.

According to Taylor’s twin cycle hypothesis, high hepatic production of a very low lipoprotein (VLDL) density rich in triglycerides, fat deposition in the liver, and overweight/obesity drive the pathogenesis of T2DM by leading to the accumulation of ectopic pancreatic fat, increased insulin resistance, and β-cell dysfunction [52]. A recent study demonstrated that even 0.5 g of excess fat can cause serious impairments in the function of pancreatic β-cells [53]. Low-energy diets leading to a dramatic change in calorie balance and eventually weight loss could lead to the reversal of these mechanisms crucial for the development of T2DM, completely in early diabetes and to a worthwhile extent in more established disease [52,53].

Although VLCD has been shown to be safe for patients with BMI >30 kg/m^2^, it is suggested to be performed under medical supervision due to the possibility of side effects. Currently, VLCD is not recommended for people of normal weight and overweight individuals with a BMI of 27–30 kg/m^2^, and should only be reserved for those with weight-related health complications [54]. Furthermore, VLCD should not be considered for people older than 50 years due to the high risk of negative nitrogen balance and for pregnant/lactating women [55]. Therefore, although VLCD appears to be an effective remission strategy, it should be used with caution.

There are many epidemiological studies suggesting that diets containing foods rich in polyphenols (i.e., grains and soy, fruits and vegetables, olive oil, red wine, tea, and coffee) could protect against the development of T2DM [56]. However, research findings on specific compounds are still inconclusive, probably due to differences in the included populations, the duration of follow-up, and the methods for evaluating dietary intake. Furthermore, it is still unclear whether the benefit in glucose metabolism is related to the effects of individual compounds in these diets or to the interaction of different components, which seems to be the most reasonable scenario. A meta-analysis of 22 randomized trials demonstrated that dietary fiber is negatively correlated with fasting insulin and the homeostatic model for insulin resistance values, while it can significantly decrease HbA1c and fasting glucose [57]. However, its impact on body weight was not significant. Relevant mechanisms include changes in the gut microbiota, which in turn lead to improved insulin secretion mediated by the increased intestinal production of glucagon-like peptide 1 (GLP-1) [58]. Future studies are expected to shed more light on whether diets rich in polyphenols and fiber could promote diabetes remission.

Adherence to strict diets for a long period of time to maintain remission remains an ongoing challenge. Furthermore, it is well-established that weight loss is followed by the up-regulation of compensatory mechanisms that oppose additional weight loss and promote weight regain, such as alterations in energy expenditure, neuroendocrine pathways, nutrient metabolism, and gut physiology [59]. This is probably why the available data indicate that almost 50% of people who lose weight will return to their baseline state in a mean period of 4 years [60]. Table 2 presents the different dietary interventions that have been shown to be effective in inducing remission of T2DM.

### 3.3. Counseling and Behavior Change

The promotion of lifestyle change could be implemented through behavioral change techniques. The Look AHEAD trial reported that rigorous specialist-led behavioral programs can facilitate weight loss, improve cardiovascular risk factors, and increase the probability of achieving remission [66]. Successful treatment and remission of T2DM are interrelated with patient behavior. In this context, it is important that specialists encourage patient adherence to medications along with dietary and lifestyle changes. In 2019, McCombie et al. showed that weight recovery after returning to typical dietary habits could be delayed if there is structured psychological support using cognitive behavior therapy [67]. Today, digital applications have been launched to facilitate behavioral change, which can be managed by nurses, nutritionists, exercise physiologists, and other health professionals. Changing Health and Low Carb Program Health Behavior Change are some examples that have been shown to improve the effects of dietary interventions. Burner et al. showed that the integration of mobile applications is a promising approach for people with diabetes to find support and nutritional information [68]. However, more research is needed to explore the effectiveness of digital behavior interventions in helping people with T2DM achieve remission.

### 3.4. Exercise

In 2004, the World Health Organization (WHO) suggested that moderate-intensity exercise improves not only physical and mental health, but also outcomes related to T2DM, cardiovascular disease, and cancer [69]. Despite the proven benefits of physical activity, many people with diabetes cannot exercise due to several (physical, mental or social) barriers. Until now, prescribing exercise has not been an effective first-line strategy to achieve remission, due to the insufficient compliance of patients with T2DM. However, recent guidelines for physical activity recommend five sessions of moderate activity per week to reduce insulin resistance [70,71]. Furthermore, the Nutrition Practice Guidelines (NPG) recommend aerobic physical activity for more than 150 min a week, evenly divided throughout the week and without a gap of more than two consecutive days without exercise. In general, the incorporation of activity plans into the daily routine has been shown to play a key role in preventing weight regain in the long term [72].

All types of exercise lead to immediate improvements in glycemic control, including aerobic, resistance, and endurance training. Research data claimed that pre-prandial resistance training and high-intensity interval exercise are the most beneficial types of exercise in terms of improving glycemic markers [73]. The first trial that provided long-term evidence of diabetes remission with increased physical activity was the 6-year Malmo feasibility study, which showed significant reductions in glucose and insulin responses to the oral glucose tolerance test, while 54% of the participants achieved diabetes remission after 5-year follow-up [74]. These findings are consistent with those of other randomized trials with a larger number of participants and a longer follow-up period that revealed a positive effect of exercise on the remission and prevention of T2DM [75]. Furthermore, several shorter studies investigating the combination of diet-induced weight loss with intensive exercise training produced impressive results. Specifically, the rates of (partial or complete) remission ranged from 37% to 80% after 3–6 kg of weight loss over a period of 0.5 to 5 years. Compared to the DiRECT and Look AHEAD studies, these findings seem more significant [76,77,78].

In recent years, neuromuscular electrical stimulation devices (NMES) have been available, which can help sedentary populations to implement exercise. Considering that an individual can consume 2000 kcal in 6 h using an NMES system, these devices can play a role in the management and prevention of T2DM [79]. Some studies demonstrated an improvement of 0.8 ± 0.7% in HbA1c with the use of these devices compared to 0.62% in groups with conventional lifestyle interventions [80]. However, more research is needed to assess the potentially beneficial effect of such methods on diabetes remission. Table 3 summarizes studies that have investigated the efficacy of lifestyle intervention to promote diabetes remission.

### 3.5. Pharmacotherapy

#### 3.5.1. Glucose-Lowering Drugs

New antidiabetic drugs have recently been introduced into clinical practice and are expected to facilitate diabetes remission due to their combined glucose and weight lowering properties [86,87]. Sodium-glucose cotransporter-2 (SGLT2) inhibitors decrease renal glucose reabsorption by acting on the convoluted tubule of the kidney, thus inducing plasma glucose reduction regardless of insulin sensitivity or insulin secretion [88]. Furthermore, glucosuria causes calorie loss and results in a decrease in weight and visceral fat. Although SGLT2 inhibitors present only a modest glucose-lowering potency, their effects on remission induction could be amplified when used in combination with other agents. McInnes et al. used SGLT2 inhibitors in combination with basal insulin and metformin and achieved remission in 24.7% of patients compared to 16.9% of the group not treated with SGLT2 inhibitors [86].

GLP-1 belongs to a broader category of incretin hormones that act on the lower digestive system and inhibit glucagon secretion, promote insulin production, and delay gastric emptying. Several GLP-1 receptor agonists are now widely used for the management of T2DM. Semaglutide and tirzepatide, a dual analogue of GLP-1 and glucose-dependent insulinotropic polypeptide (GIP), have recently been added to the pharmaceutical arsenal against T2DM. Both molecules showed very promising results in phase 3 trials and could be considered game changers in the pursuit of remission. Tirzepatide resulted in impressive remission rates ranging from 66% to 81% after 52 weeks dependent on the drug dosage. Furthermore, a study showed that 51.7% of the individuals treated with tirzepatide achieved an HbA1c of 5.7% and an average weight loss of 9.5 kg [89]. These drugs rarely cause hypoglycemic events, while the most common adverse events related to their use are gastrointestinal disorders (such as nausea, diarrhea, and vomiting) [90]. Regarding semaglutide, studies showed an average weight reduction of 10 kg together with an average HbA1c of 6.4% with its use [91]. Vadher et al. reported that HbA1c and weight reduction were significantly higher in participants who received the highest doses of tirzepatide (10 mg and 15 mg) than in those who received 2 mg of semaglutide. On the contrary, the results were similar between the tirzepatide 5 mg and semaglutide 2 mg groups [92]. Future trials will show whether emerging pharmacological treatments, such as incretin-based triagonists, can be equally effective as metabolic surgery in promoting diabetes remission.

Metformin has been used for the treatment of diabetes for more than six decades, having an excellent safety and efficacy profile. Although metformin exhibits a wealth of pleiotropic actions that positively affect cardiovascular disease risk factors, such as the lipid profile, its weight reduction potential is weak, while evidence for cardioprotection with metformin is mostly observational [93]. In this context, combination therapies consisting of metformin and other glucose-lowering drugs could lead to higher remission rates compared to those achieved by various agents alone. Sugiyama et al. recently reported a case of a patient who completely recovered from T2DM after treatment with the SGLT2 inhibitor dapagliflozin and metformin, accompanied by an impressive reduction in baseline body weight [94]. A study showed a positive effect in terms of remission of diabetes using a triple combination of metformin, pioglitazone, and repaglinide at the maximum tolerated doses [95]. Another drug combination (metformin, gliclazide, pioglitazone) was effective in inducing remission of T2DM in less than two years, also decreasing insulin requirements [96]. Furthermore, intensive insulin use for a short period of time (2 or 3 weeks) can improve β-cell function, leading to remission in 46% of patients in one year, regardless of weight loss [97]. The response to various treatments may be heterogeneous, depending on the genetic, metabolic, and phenotypic characteristics of each patient. Furthermore, each antidiabetic drug has different effects on glycemia and body weight that physicians must consider when building the therapeutic regimen. For example, insulin, pioglitazone, glinides, and sulfonylureas have a strong glucose-lowering potency but tend to increase body weight, so they may not be ideal agents for promoting remission. Moreover, sulfonylureas have been associated with secondary treatment failure, which in the long term might jeopardize the achievement of remission. Therefore, metformin, SGLT2 inhibitors, GLP-1 receptor agonists, and tirzepatide should be prioritized in most patients due to their ability to provide a sustainable glucose reduction effect with a low risk of hypoglycemia and facilitate weight loss.

#### 3.5.2. Anti-Obesity Drugs

Weight loss is clinically important not only because it can promote remission in patients with T2DM, but also because it has been associated with improvements in weight-related complications such as cardiovascular disease [98]. The Food and Drug Administration (FDA) has approved five medications for chronic weight management: orlistat, lorcaserin, phentermine/topiramate, bupropion/naltrexone, and liraglutide. In the SEQUEL trial, obese patients with T2DM who received phentermine/topiramate 15/92 mg once daily showed changes in insulin, fasting glucose, and HbA1c levels after weight loss of 10% or more, compared to placebo [99]. Another anti-obesity drug, orlistat, which has been available for more than two decades, improves the glycemic profile. However, gastrointestinal side events cause poor patient compliance [100]. Furthermore, orlistat selectively reduces visceral fat and prevents the digestion of free fatty acids, which are responsible for the increase in hepatic and peripheral insulin resistance [101], and increases the secretion of two gut hormones, GLP-1 and GIP, thus improving insulin release [102]. In 2005, a meta-analysis of seven randomized control trials showed that patients who received orlistat at a dose of 120 mg three times a day had an average weight loss of 3.9% after 12 weeks compared to 1.44% in the placebo group [103]. Recently, Ardissino et al. published the results of a propensity-score matched cohort study that included 36,876 patients with obesity, showing that orlistat use was associated with a lower risk of major adverse cardiovascular events (MACE), new-onset heart failure, renal damage, and mortality [104].

Lorcaserin is a selective serotonin 2C receptor agonist that acts on serotonin receptors in anorexigenic proopiomelanocortin (POMC) neurons in the hypothalamus and increases satiety and reduces caloric intake [105]. In 2018, Bohula et al. showed that the drug has the potential to mitigate the risk of incident diabetes, induce remission, and reduce the risk of microvascular complications in obese and overweight patients [106]. According to the BLOOM-DM trial, a weight loss of at least 5% of baseline body weight was achieved in 44.7% and 37.5% of patients with T2DM who received 10 mg of lorcaserin once and twice daily, respectively, compared to 16.1% in the placebo group. Furthermore, lorcaserin was associated with a statistically significant decrease in HbA1c, specifically 0.9%, 1.0% and 0.4% in the once daily, twice daily and placebo groups, respectively [107]. Lorcaserin has been shown to facilitate weight loss without increasing the risk of cardiovascular events [108], while the rates of valvulopathy, depression, and suicidal risk do not differ between lorcaserin users and those receiving placebo [109].

Eight years ago, the FDA approved the combination of bupropion, which is a dopamine and norepinephrine reuptake inhibitor, and naltrexone, an opioid receptor antagonist. This combination acts by increasing the stimulation of anorexigenic POMC neurons and the release of anorectic alpha-melanocyte stimulating hormone [110]. In the COR-Diabetes trial, patients treated with bupropion/naltrexone had a two-fold higher percentage of weight loss compared to the placebo group (44.5% vs. 18.9%) and a six-fold reduction in HbA1c (0.6% vs. 0.1%) [111]. Common adverse events of this combination include nausea, headache, and constipation, which are dose-dependent, while its use has been shown to be safe with respect to the risk of MACE [112]. The GLP-1 receptor agonist liraglutide has been approved for the treatment of T2DM at a dose of 1.8 mg daily and for chronic weight management at a dose of 3.0 mg daily [113]. The most frequent adverse events seen in patients treated with GLP-1 receptor agonists are gastrointestinal disorders, such as nausea, diarrhea, and constipation, which are, in most cases, transient. In the LEADER trial, liraglutide significantly reduced the risk of MACE, cardiovascular death, and all-cause mortality [114]. The SCALE-diabetes randomized clinical trial demonstrated weight loss of 6%, 4.7%, and 2% in patients who received 3.0 mg and 1.8 mg of liraglutide and the placebo, respectively. In addition to weight loss, the decrease in HbA1c was 1.3%, 1.1%, and 0.3% in the respective groups [115]. The aforementioned anti-obesity drugs also have a favorable impact on secondary cardiovascular endpoints, especially blood pressure, heart rate, lipoproteins, and triglycerides. Table 4 summarizes the studies investigating the efficacy of anti-obesity drugs in people with T2DM.

## 4. Below the Surface: Remission Effects on Different Tissues

In addition to diabetes, nonalcoholic fatty liver disease (NAFLD) has become a major public health concern. NAFLD, which is strongly associated with T2DM, is one of the main causes of liver-related morbidity and mortality and plays a key role in the progression of metabolic diseases. Furthermore, the risk of NAFLD is higher in patients with diabetes, closely related to the development of complications, such as cardiovascular disease. Tirzepatide has been shown to significantly reduce liver fat content, as assessed by magnetic resonance imaging in people with T2DM [119]. Although studies that evaluate its effects on liver histology are currently lacking, the impressive reduction in weight loss induced by the dual agonist sets the stage for the introduction of a new player in the treatment of NAFLD. A recent meta-analysis of eight randomized trials showed that compared to placebo, GLP-1 receptor agonist significantly improved biopsy resolution in patients with T2DM and NAFLD, in addition to biochemical markers of liver function [120]. Whether the benefits of incretin-based therapies in NAFLD and steatohepatitis are exclusive to weight loss or mediated by the inflammatory actions of these drugs remains an area of future research.

The pancreas is known to be smaller and has an irregular shape in overweight individuals with diabetes, and this could be explained by the loss of the paracrine action of insulin and the high secretion of fibroblast growth factors (especially FGF-21, FGF-19), which cause fat accumulation within the pancreas [121]. This fat causes fibrosis of acinar cells and is potentially related to a decrease in the volume of the pancreas. Therefore, moderate weight loss can break the vicious cycle and improve liver steatosis, insulin resistance, and hyperglycemia [122]. A post hoc analysis of the DiRECT trial showed changes in the gross morphology of the pancreas after 2 years of follow-up following remission of T2DM [123]. The volume of the pancreas increased in patients who achieved remission and weight loss compared to those who did not respond to the weight loss intervention. Endopancreatic fat and FGF-21 and FGF-19 levels also decreased. While β-cell damage is crucial for the development of T2DM, several experimental studies have provided preliminary evidence that remission could restore β-cell function. The exact mechanisms are still under investigation, but they are believed to be related to the alleviation of endoplasmic reticulum stress, improved mitochondrial function, favorable changes in gene expression in pancreatic islets, the amelioration of pancreatic inflammation, and the down-regulation of glucolipotoxicity and metabolic stress, among others [124]. Thus, mitigating β-cell dysfunction appears to be a key target in the effort to achieve long-term diabetes remission.

Sarcopenic obesity is a clinical entity that is gaining increasing attention and is characterized by the combination of low muscle mass and increased fat mass. There are not enough data to draw definite conclusions about the effects of remission on skeletal muscle. Martinez et al. reported a significant muscle mass loss maintained 24 months after metabolic surgery, despite the dramatic improvement in insulin resistance within one month after the procedure [125]. Recently, Nguyen et al. found that in male patients with T2DM who underwent laparoscopic sleeve gastrectomy, the remission rate increased by 26% for each additional percentage of gain in skeletal muscle 12 months after the procedure [126]. Several studies have shown that bariatric surgery promotes changes in adipose cell morphology, including increased lipolysis pathways and hyperplasia of adipose cells and a decrease in size, which together contribute to the down-regulation of insulin resistance and the improvement in glycemic markers seen after surgery [127]. More studies are needed to provide a deeper understanding of the relationship between diabetes remission and changes in the various tissues involved in glucose homeostasis.

## 5. In Pursuit of Remission: Benefits and Challenges

The available data suggest that T2DM can be reversed; however, the critical question is whether remission can prevent long-term macrovascular and microvascular damage. An unequivocal answer can only be given from studies with a long follow-up period after the achievement of remission. Remission early after diagnosis appears to reduce the subsequent risk of complications, a phenomenon described as “metabolic memory” or “legacy effect”. This is applicable primarily to microvascular complications and secondary to macrovascular disease, and therefore people with diabetes require continuous surveillance for recurrence and complications even after remission is achieved [121]. According to long-term observational studies, bariatric surgery was associated with high rates of remission and lower rates of microvascular and macrovascular complications in the long term, as well as mortality [128,129]. However, different studies have shown that bariatric surgery cannot prevent diabetic neuropathy, nephropathy, albuminuria, and retinopathy [130,131,132].

Bariatric surgery and by extension weight loss can improve risk factors for cardiovascular disease, such as blood pressure and serum lipid levels. Furthermore, several studies have reported that weight loss through lifestyle modification has beneficial effects on common diabetes and obesity comorbidities, including cancer, osteoarthritis, chronic kidney disease, infertility, and sleep apnea, and reduces the economic burden on health care systems [133,134]. In addition, a diagnosis of diabetes can have a negative impact on the mental health of patients and, consequently, on the self-management of their condition. On the contrary, lifestyle changes and weight loss can have a positive effect on mood and quality of life. A study in the UK, including participants who achieved remission of T2DM, showed that 38% experienced a beneficial effect on mood status, 26% reduced their antidiabetic medications, and 51% achieved an improvement in HbA1c levels [48].

Despite the aforementioned benefits, remission of diabetes according to its definition a priori requires the interruption of any anti-diabetic medication. During the last decade, the introduction of SGLT2 inhibitors and GLP-1 receptor agonists into daily practice has revolutionized the management of T2DM. Both classes of drugs have shown remarkable cardiorenal benefits in large-scale cardiovascular outcome trials, in addition to effectively lowering blood glucose with minimal risk of hypoglycemia. More specifically, SGLT2 inhibitors have been shown to reduce the risk of MACE and hospitalization for heart failure, slow the progression of kidney disease, and reduce cardiovascular and all-cause mortality in people with heart failure and renal impairment, regardless of diabetes status [135]. Of great importance, these benefits appear to be evident within the first weeks of treatment [136]. On the other hand, GLP-1 receptor agonists have been shown to reduce the risk of MACE, macrovascular endpoints such as stroke and myocardial infarction, and cardiovascular death in people with T2DM and established cardiovascular disease [137]. Furthermore, accumulating data suggest that both categories manifest a wealth of pleiotropic actions that can improve outcomes beyond the spectrum of diabetes, such as cognitive impairment, infections, and cancer [138,139]. In this context, recent guidelines for the treatment of T2DM advocate the use of these agents in patients with established complications or who are at high risk, regardless of the quality of glycemic control, to take advantage of their cardiorenal and weight-lowering benefits that appear to be independent of their glucose-lowering action [140]. This is particularly important, considering that strict glycemic control with conventional agents such as insulin or sulfonylureas has been shown to reduce the risk of microvascular disease; however, its impact on macrovascular outcomes needs enough time to become evident, while whether it can reduce cardiovascular and all-cause mortality rates remains controversial [141]. Taking into account the above, the decision to stop treatment with agents that have proven cardiorenal benefits, especially among patients at high risk, needs careful consideration.

Although previous studies have identified specific clinical and laboratory predictors of remission, such as duration of diabetes, baseline weight, burden of comorbidities, age, and c-peptide levels, a challenge for future research is to better define these factors and incorporate them into clinical management algorithms that will be available and easy for clinicians to use in daily practice [142]. As the importance of remission increases, specialists and society must focus on the pandemic of lifestyle diseases and provide evidence-based guidance to people with T2DM to help them achieve it. This will require clinicians to integrate multivariate approaches to diabetes management and public health authorities to implement policies that promote a healthy lifestyle in all aspects of social and public life.

## 6. Conclusions

The increasing incidence of diabetes is an emerging global concern. This trend appears to be closely related to the aging of the population in combination with unfavorable changes in physical activity and eating patterns, resulting in high rates of obesity. However, there is evidence that in the initial stages after diagnosis, significant weight loss with surgical or dietary approaches can induce remission of diabetes at a rate greater than 50%. At the same time, new medications, such as SGLT2 inhibitors and GLP-1 analogues or their combination, have a secondary weight loss effect, which can lead several patients to remission with minimal risk of hypoglycemia. Preliminary data support the notion that remission can prevent complications associated with diabetes and reduce the burden of the disease on health care systems and societies. However, maintaining long-term remission requires continuous medical supervision and support from healthcare providers through a personalized approach. Considering that remission has only recently emerged as a treatment goal in T2DM, several aspects, including the ideal percentage of weight loss to be targeted and the long-term impact on health economics, remain to be clarified by future studies. Furthermore, more research is needed to determine the optimal lifestyle, pharmacological, or surgical approaches that will help patients with diabetes achieve and maintain remission.

## Figures and Tables

**Table 1:** Studies that have investigated the efficacy of metabolic surgery in inducing remission.

Study	Study Description	Study Design	Intervention Methods	Follow-Up	Remission/Findings
Iaconelli A et al., 2011 [38]	*n* = 110, BMI > 35 kg/m^2^	Open-case control	BPD vs. medical treatment	10 years	↑T2DM remissions observed in all BPD patients, ↓microvascular complications
Mingrone et al., 2012 [34]	*n* = 20 vs. *n* = 20, BMI ≥ 35 kg/m^2^	RCT	BPD versus RYGB	10 years	95% vs. 75%, HbA1c < 6.5%, weight loss 33.8% vs. 33.3%
Courcoulas et al., 2014 [39]	*n* = 21 vs. *n* = 20, BMI 30–40 kg/m^2^	RCT	RYGB versus AGB	12 months	27% vs. 23%, HbA1c < 5.7%, weight loss 27.0% vs. 17.3%
Arterburn et al., 2013 [40]	*n* = 4434, BMI > 35 kg/m^2^	Retrospective cohort	RYGB, sleeve gastrectomy, AGB	10 years	76.9% partial remission, 68.2% complete remission among all patients, HbA1c < 6.5%
Schauer et al., 2012 [41]	*n* = 50 vs. *n* = 50, BMI 27–43 kg/m^2^	Randomized, non-blind, single-center trial	RYGB versus SG	12 months	42% vs. 37%, HbA1c < 6.0%, weight loss 29.4 kg vs. 25.1 kg
Cummings et al., 2016 [42]	*n* = 23 vs. *n* = 20, BMI 30–45 kg/m^2^	RCT	RYGB versus medical treatment	12 months	60% vs. 6%, HbA1c < 6.0%, weight loss 25.8% vs. 6.4%
Schauer et al., 2017 [36]	*n* = 150, BMI 27–43 kg/m^2^	RCT	RYGB or sleeve gastrectomy versus medical treatment	5 years	Remission not mentioned, ↓HbA1c 2.1% vs. 0.3%, weight loss 23%, 19% vs. 5%
Courcoulas et al., 2018 [43]	*n* = 1738 vs. *n* = 610, BMI 44–47 kg/m^2^	Observational study	RYBG versus LABG	7 years	60.2% vs. 20.3%, weight loss 38.2 kg vs. 18.8 kg
Jakobsen et al., 2018 [44]	*n* = 932 vs. *n* = 956, median BMI 44.2 kg/m^2^	Registry-based cohort study	RYGB or sleeve gastrectomy versus medical treatment	7 years	57.5% vs. 14.8%, ↓risk for complications, ↓risks of obesity-related Comorbidities
Young et al., 2019 [37]	*n* = 75 vs. *n* = 26, median BMI 43.1 kg/m^2^	Cohort Study	RYGB versus sleeve gastrectomy	10 years	↑T2DM remission, HbA1c ≤ 6.7%, ↓BMI and ↓albuminuria
McGlone et al., 2020 [45]	*n* = 1847 (surgical group), median BMI 47.2 kg/m^2^	Retrospective study	RYGB, ABG, sleeve gastrectomy versus medical treatment	5 years	Surgical group: no indication of T2DM, 36.6%, total weight loss, 27.1%
Mingrone et al., 2021 [35]	*n* = 60, BMI ≥ 35 kg/m^2^	RCT	RYGB, BPD versus medical treatment	10 years	Surgical group vs. medical treatment, 37.5% vs. 5.5%, remission, ↓ complications of diabetes

T2DM: type 2 diabetes mellitus, HbA1c: glycosylated hemoglobin, BMI: body mass index, RYGB: Roux-en-Y gastric bypass, AGB: adjustable gastric band, SG: sleeve gastrectomy, BPD: bilopancreatic diversion, ↑ increased, ↓ decreased.

**Table 2:** Studies that have investigated the efficacy of dietary interventions in inducing remission.

Study	Study Description	Study Design	Diets Intervention	Follow-Up	Weight Loss	Remission Rate, Outcomes
Lim et al., 2011 [47]	*n* = 11 vs. *n* = 8, BMI 25–45 kg/m^2^	Counterpoint study	VLCD versus medical intervention	2 months	13.1 kg	100%, fasting plasma glucose < 126 mg/dL
Steven et al., 2015 [61]	*n* = 15 vs. *n* = 14, BMI 27–45 kg/m^2^	Single-arm intervention study	VLCD, T2DM of < 4 years versus T2DM of > 8 years	2 months	14.8 kg vs. 14.4 kg	87% vs. 50%, HbA1c < 6.5%
Steven et al., 2016 [62]	*n* = 15 vs. *n* = 14, BMI 27–45 kg/m^2^	Single-arm intervention study	VLCD, T2DM of < 4 years versus T2DM of > 8 years	6 months	15.8 kg vs. 13.5 kg	Total remission 43%, HbA1c < 6.5%
Lean et.al.,2018 [48]	*n* = 149 vs. *n* = 149, BMI 27–45 kg/m^2^	RCT	VLCD (intervention vs. control group)	1 year	10 kg vs. 1 kg	46% vs. 4%, HbA1c < 6.5%
Lean et.al., 2019 [18]	*n* = 149 vs. *n* = 149, BMI 27–45 kg/m^2^	RCT	VLCD (intervention vs. control group)	2 years	7.5 kg vs. 2.3 kg	36% vs. 3%, HbA1c < 6.5%
Pan et al., 2019 [63]	Ten RCTs	Meta-analysis	Mediterranean diet	-	Beneficial ↓ in weight loss, waist circumference and a significant ↓ of HbA1c compared to regular diet	
Umphonsathien et al. 2019 [64]	*n* = 19, BMI 23–30 kg/m^2^	Single-arm intervention study	VLCD	14 weeks	9.5 kg	79%, HbA1c < 6.5%, fasting plasma glucose < 126 mg/dL
Taheri et al., 2020 [65]	*n* = 70 vs. *n* = 70, BMI > 27 kg/m^2^	RCT	VLCD versus medical intervention	1 year	12 kg vs. 4 kg	61% vs. 12%, HbA1c < 6.5%

T2DM: type 2 diabetes mellitus, HbA1c: glycosylated hemoglobin, BMI: body mass index, FPG: fasting plasma glucose, VLCD: very low-calorie diet, ↓ decrease.

**Table 3:** Studies that have investigated the efficacy of combined lifestyle interventions to promote diabetes remission.

Study	Study Description	Study Design	Interventions	Follow-Up	Outcomes
Maraki et al., 2011 [81]	*n* = 16, obese and lean men	Prospective, matched, controlled study	Diet, physical activity	15 weeks	Weight loss 10 %, ↓ plasma glucose and serum insulin concentrations
Shantha et al., 2012 [82]	*n* = 121, BMI ≥ 25 kg/m^2^	Retrospective, uncontrolled cohort study	Calorie-restricted diet, behavior modification, increased physical activity	Mean 13.2 months	Weight loss, 7.8 %, for each 10% weight loss, ↓ HbA1c of 0.81%
Shantha et al., 2013 [83]	*n* = 179, BMI ≥ 25 kg/m^2^	Retrospective, uncontrolled cohort study	Calorie-restricted diet, behavior modification, increased physical activity	15 months	Weight loss, 12.2%, mean ↓ HbA1c of 0.5%
Imayama et al., 2013 [84]	BMI ≥ 25 kg/m^2^ Asian and ≥ 23 kg/m^2^ American	Randomized controlled trial	Diet and/or exercise versus control	12 months	Weight loss in diet + exercise group, 10.8%, improvements in insulin, c-peptide, glucose levels
Golubic et al., 2018 [85]	*n* = 141, BMI ≥ 40 kg/m^2^	Prospective, uncontrolled, cohort study	Dietary interventions, pharmacotherapy, physical activity, and behavior modification	3 months	Weight loss, 15%, mean ↓ HbA1c of 0.6%

HbA1c: glycosylated hemoglobin, BMI: body mass index, ↓ decreased.

**Table 4:** Studies investigating the efficacy of antiobesity drugs in people with T2DM.

Drug	Action	Route and Dose	Weight Loss at 1 Year	HbA1c < 6.5% in Drug vs. Placebo Group
Orlistat [101]	Inhibitor of gastrointestinal lipase	Oral—120 mg three times a day	2.5%	Not mentioned.
Phentermine-topiramate [116]	Central norepinephrine release	Oral—15 mg of phentermine/92 mg of topiramate once a day	6.9%, 6.7%	37% vs. 17%, 32% vs. 16%
Naltrexone–bupropion [117]	Increased central norepinephrine and dopamine and opioid receptor antagonist	Oral—16 mg naltrexone/180 mg bupropion twice a day	3.2%	20.7% vs. 10.2%
Liraglutide [115]	GLP-1 agonist	Subcutaneously—3 mg once a day	4.0%	56.5% vs. 15.0%
Lorcaserin [107]	Selective serotonin 2C receptor agonist	Oral—10 mg once or twice a day	2.6 kg diabetic and 2.8 kg in prediabetic patients	33.9% vs. 8%
Semaglutide 2.4 mg [118]	GLP-1 agonist	Subcutaneously—2 or 4 mg once per week	6.2%	67.5% vs. 15.5%
Tirzepatide 5, 10, 15 mg [92]	GLP-1 and GIP analog	Subcutaneously—5, 10 or 15 mg once a week	7.0 kg, 7.8 kg, 9.5 kg	↓HbA1c: −1.87% (5 mg), −1.89% (10 mg), −2.07% (15 mg), +0.04% (placebo)

HbA1c: glycosylated hemoglobin, GLP-1: glucagon-like peptide-1 analogs, GIP: glucose-dependent insulinotropic polypeptide, ↓ decreased.

## Data Availability

Not applicable.
